# High-Performance Computing and Big Data in Omics-Based Medicine

**DOI:** 10.1155/2014/825649

**Published:** 2014-12-22

**Authors:** Ivan Merelli, Horacio Pérez-Sánchez, Sandra Gesing, Daniele D'Agostino

**Affiliations:** ^1^Bioinformatics Research Unit, Institute for Biomedical Technologies, National Research Council of Italy, Segrate (MI), Italy; ^2^Bioinformatics and High Performance Computing Research Group, Computer Science Department, Universidad Católica San Antonio de Murcia (UCAM), 30107 Murcia, Spain; ^3^Department of Computer Science and Engineering, Center for Research Computing, University of Notre Dame, Indiana, USA; ^4^Advanced Computing Systems and High Performance Computing Group, Institute for Applied Mathematics and Information Technologies, National Research Council of Italy, Genoa, Italy

Omics sciences are able to produce, with the modern high-throughput techniques of analytical chemistry and molecular biology, a huge amount of data. Next generation sequencing (NGS) analysis of diseased somatic cells, genome wide identification of regulatory elements and biomarkers, systems biology studies of biochemical pathways and gene network, and novel bioactive compounds discovery and design are examples of the great advantages that high-performance computing (HPC) can provide to omics sciences, providing a fast translation of biomolecular results to the clinical practice.

Massive parallel clusters, distributed technologies such as grid and cloud computing, and on-chip supercomputing such as GPGPU and Xeon Phi devices represent well-established solutions in research laboratories, but their capabilities should be widespread by clinical and healthcare experts to reach their full potential. Additionally to compute-intensive applications, health care problems are concerned with data-intensive applications. Managing terabytes of data requires specific technologies such as redundant facilities, shared and distributed file systems, clustered databases, indexing and searching processes, and dedicated network configuration.

The aim of this special issue is to present the latest advances in the field of omics data processing with high-performance computing solutions and big data analysis paradigms, showing the potential repercussions of these technologies in translational medicine. In particular, a review paper, authored by the editors, “*Managing, analysing and integrating big data in medical bioinformatics: open problems and future perspectives*,” is a survey about open problems and future perspectives in the management, analysis, and integration of big data in medical bioinformatics, while the eleven selected research papers present novel concepts and important results about the application of HPC techniques in the field of computational and systems biology.

Regarding the research papers, three contributions propose novel or improved implementation of parallel algorithms. C. Misale et al. in their paper entitled “*Sequence alignment tools: one parallel pattern to rule them all?*” are engaged in NGS and investigated the performance of the widely used alignment tools Bowtie2, BWA, and BLASR. They advocate high-level parallel programming as an alternative design strategy for next generation alignment tools. The master-worker FastFlow pattern to Bowtie2 and BWA-MEM increases the speedup for both applications and is compared to manually tuned implementations. The paper by M. W. Al-Neama et al. “*An Improved distance matrix computation algorithm for multicore clusters*” is concerned with an efficient implementation of distance matrix computation on multicore clusters that finds wide application in multiple sequence alignment. The implementation is mainly based on MPI and OpenMP libraries and achieves improved speedups with respect to the public parallel implementation of ClustalW-MPI. M. Aldinucci et al. in the paper entitled “*On designing multicore-aware simulators for systems biology endowed with online statistics*” describe a multicore aware simulation algorithm that can be applied in the field of systems biology for online statistics and data mining. The main methodology is based on the acceleration of the simulation of stochastic models and the analysis of the obtained results, where the application case relies on a general framework for the modelling of biological systems and their stochastic behaviour called calculus of wrapped compartments.

The paper by P. Cazzaniga et al. “*Massive exploration of perturbed conditions of the blood coagulation cascade through GPU parallelization*” belongs to the class of research on on-chip acceleration of the behaviour of biological systems in different conditions. The paper is concerned with GPU implementation of a deterministic systems biology simulator, which is applied for the exploration of perturbed conditions of the blood coagulation cascade using GPU hardware. It is based on the automatic derivation of the system of ordinary differential equations that represent the blood system in parallel. Most interesting results are obtained by one-dimensional and two-dimensional sweep of simulation parameters and achieved speedup is around 181x.

Three papers discuss approaches based on the combined use of the parallel/distributed computing with the usage of GPU-based devices. The paper by G. D. Guerrero et al. “*A performance/cost evaluation for a GPU-based drug discovery application on volunteer computing*” discusses the benefits of volunteer computing to scale bioinformatics applications as an alternative to own large GPU-based local infrastructures. They used as benchmark a GPU-based drug discovery application called BINDSURF and the Ibercivis, which relies on BIOINC, as the reference platform. D. D'Agostino et al. in the paper entitled “*Parallel solutions for voxel-based simulations of reaction-diffusion systems*” present a comparison between MPI and a GPU implementation of a stochastic simulator able to consider both time and space in its simulations. This algorithm is also crowd-aware and it has been tested on a gene regulatory network, whose behaviour is influenced by both the small number of molecules involved and the conformation of the DNA in the nucleus. The paper by J. Colmenares et al. entitled “*A combined MPI-CUDA parallel solution of linear and nonlinear Poisson-Boltzmann equation*” is on a combined MPI/CUDA approach for the solution of linear and nonlinear Poisson-Boltzmann equation, which is very relevant to modeling the electrostatic potential generated by a system of charged particles immersed in an ionic solution. Their work is mainly based on the implementation of a full Poisson-Boltzmann solver based on a finite-difference scheme on a heterogeneous parallel system comprised of a cluster of multicores and multi-GPUs. Overall speedups of around 20x are achieved.

Two papers investigate computational biology problems that present scalability issues in their real-life application. The paper by M. Lauria “*Rank-based miRNA signatures for early cancer detection*” describes a new signature definition and analysis method to be used as biomarker for early cancer detection. This approach is based on the construction of a reference map of transcriptional signatures of both healthy and cancer affected individuals using circulating miRNA from a large number of subjects. T. Garcia-Valverde et al. in the paper entitled “*Heart health risk assessment system: a nonintrusive proposal using ontologies and expert rules*” suggest ontology and expert rules for creating a heart health risk assessment system. Available knowledge about persons gained by sensors embedded in smartphones and smartwatches allows for different levels of alerts or suggestions for the users when the intensity of the activity is detected as dangerous for their health.

Finally, two papers discuss distributed approaches to large-scale computation. J. Krüger et al. in the paper entitled “*Performance studies on distributed virtual screening*” elaborate the performance for virtual high-throughput screening in regard to distributed computing on the example of UNICORE. They considered not only the runtime of the docking itself, but also the effort required for structure preparation. Performance studies were conducted via the workflow-enabled science gateway MoSGrid (Molecular Simulation Grid). A. Ragothaman et al. in the paper entitled “*Developing eThread pipeline using SAGA-pilot abstraction for large-scale structural bioinformatics*” present a cloud infrastructure for running eThread, a metathreading protein structure modelling tool for structural bioinformatics, using Amazon EC2. Authors supplied interesting insights of how continuing advances in genome sequencing technologies result in rapidly decreasing costs of experiments making them affordable for small research groups. Moreover, they present how it is possible to exploit modern cloud infrastructures to execute large-scale calculations by coordinating possibly heterogeneous virtual machine instances.

*Ivan Merelli* *Horacio Pérez-Sánchez* *Sandra Gesing* *Daniele D'Agostino*

